# Pre-Pulseless Takayasu Arteritis is Associated with Distinct Clinical and Angiographic Features but Similar Outcomes – A Cohort Study

**DOI:** 10.31138/mjr.301223.ppt

**Published:** 2023-12-30

**Authors:** Durga Prasanna Misra, Upendra Rathore, Swapnil Jagtap, Prabhaker Mishra, Darpan R Thakare, Kritika Singh, Tooba Qamar, Deeksha Singh, Juhi Dixit, Manas Ranjan Behera, Neeraj Jain, Manish Ora, Dharmendra Singh Bhadauria, Sanjay Gambhir, Vikas Agarwal, Sudeep Kumar

**Affiliations:** 1 Department of Clinical Immunology and Rheumatology, Sanjay Gandhi Postgraduate Institute of Medical Sciences (SGPGIMS), Lucknow, India,; 2 Department of Biostatistics and Health Informatics, Sanjay Gandhi Postgraduate Institute of Medical Sciences (SGPGIMS), Lucknow, India,; 3 Department of Nephrology, Sanjay Gandhi Postgraduate Institute of Medical Sciences (SGPGIMS), Lucknow, India,; 4 Department of Radiodiagnosis, Sanjay Gandhi Postgraduate Institute of Medical Sciences (SGPGIMS), Lucknow, India,; 5 Department of Nuclear Medicine, Sanjay Gandhi Postgraduate Institute of Medical Sciences (SGPGIMS), Lucknow, India,; 6 Department of Cardiology, Sanjay Gandhi Postgraduate Institute of Medical Sciences (SGPGIMS), Lucknow, India

**Keywords:** Takayasu arteritis, aortic arch syndromes, outcome assessment, pre-pulseless, systemic vasculitis

## Abstract

**Objectives:**

To compare the presentation, angiographic features, evolution, and prognosis of prepulseless Takayasu arteritis (TAK) with TAK with pulse loss.

**Methods:**

Pre-pulseless TAK (defined as without pulse loss in the upper limbs, lower limb, carotid, or subclavian arteries) were identified from a cohort of TAK. Demographic characteristics, clinical features, angiographic involvement, baseline and longitudinal patterns of disease activity, medication use, and mortality rates were compared between pre-pulseless TAK and TAK with pulse loss. Adjusted odds ratios (aOR, with 95%CI) for categorical variables between pre-pulseless TAK and TAK with pulse loss were computed using multivariable-adjusted logistic regression models. Time-to-event data was compared using hazard ratios (HR) with 95%CI.

**Results:**

Compared with TAK with pulse loss, pre-pulseless TAK (91/238, 38.24%) more frequently had deranged renal function (aOR 4.43, 95%CI 1.58–12.37) and Hata’s type IV disease (aOR 8.02, 95%CI 2.61–24.65), and less often had pulse or blood pressure asymmetry (aOR 0.34, 95%CI 0.18–0.63), limb claudication (aOR for upper limb 0.38, 95%CI 0.18–0.82, for lower limb 0.28, 95%CI 0.12–0.68), right subclavian (aOR 0.45, 95%CI 0.23–0.90) or left carotid artery involvement (aOR 0.42, 95%CI 0.21–0.84). Only two patients with pre-pulseless TAK developed pulse loss on follow-up. Despite fewer pre-pulseless TAK having active disease at presentation, similar proportions of patients in both groups had active disease on follow-up. Survival was similar in both groups (HR for mortality 0.41, 95%CI 0.09–1.90).

**Conclusion:**

Pulse loss on follow-up is uncommon in those with prepulseless TAK. Pre-pulseless TAK is associated with similar long-term outcomes to TAK with pulse loss.

## INTRODUCTION

Takayasu arteritis (TAK) is an uncommon large vessel vasculitis (LVV) characterised by granulomatous aortic and arterial inflammation. Arterial wall stenosis leading to pulse loss is more often seen in TAK than in Giant Cell Arteritis, the other variant of LVV, where arterial dilatation is more frequent.^1–3^. Therefore, TAK is often referred to as “pulseless disease”.^1^ Diminution of peripheral pulses is included in classification criteria for both adult-onset and paediatric-onset TAK.^4–6^ Pulse loss is also included in scoring systems to assess disease activity and extent in TAK.^7^

It is believed that TAK progresses through three phases, initially the pre-pulseless phase with constitutional symptoms, then with features of arterial inflammation, and finally arterial stenosis with decreased or absent pulses. However, a recent multicentric study from the United States of America reported that such a triphasic pattern of disease was seen in only one-fifth of TAK.^8^ Data from large series of TAK suggests that over one-third do not have pulse loss at diagnosis.^6,9^ However, differences in the presentation, angiographic features, and prognosis (including the evolution) of pre-pulseless TAK and TAK with pulse loss have not been systematically studied. In this study, we compared the demographic and clinical features, angiographic involvement, longitudinal patterns of disease activity and treatment, and prognosis of pre-pulseless TAK and TAK with pulse loss at baseline from a large cohort from India.

## MATERIALS AND METHODS

Information was retrieved from an ambispective monocentric cohort of TAK at a tertiary care Rheumatology centre in North India.^10–13^ Patients with TAK were prospectively enrolled into the cohort from January 2023 after seeking written informed consent (ethics approval document 2022-152-IMP-129 dated 12 January 2023) and clinical details from earlier visits were retrospectively collected using standardised case record forms. Information for patients seen from 2017–2023 without visits thereafter was retrieved from vasculitis clinic files (waiver of written informed consent granted by the Institute Ethics Committee, ethics approval document 2023-34-IMP-EXP-51 dated 03 April 2023). The included patients fulfilled the 1990 American College of Rheumatology (ACR),^4^ the 2022 ACR-European Alliance of Associations for Rheumatology (EULAR) classification criteria, ^6^ or the 2012 Chapel Hill Consensus Conference definitions for TAK ^14^ (for adult-onset TAK) or the EULAR/Paediatric Rheumatology International Trials Organisation/Paediatric Rheumatology European Society classification criteria (for paediatric-onset TAK, i.e., disease onset ≤18 years of age).^5^ Pre-pulseless TAK was defined as TAK without pulse loss in the upper limbs, lower limbs, carotid, or subclavian arteries. Patients with diminished or asymmetric pulses without pulse loss were categorised under pre-pulseless TAK.

Standardised case record forms were filled from the data recorded in the clinic files to record demographic details, clinical features, angiographic involvement at presentation (including Hata’s angiographic subtype),^15^ and medication use. Disease activity at baseline using physician global assessment (PGA) of disease as active or inactive, Indian Takayasu Clinical Activity Score (ITAS2010), Disease Extent Index in TAK (DEI.TAK),^7^ and PGA at follow-up visits at 6 months, 1 year, 2 years, 5 years, and 10 years were recorded. In those with pre-pulseless TAK, whether arterial pulses had been subsequently lost was noted from the clinic files. Survival was estimated using Kaplan-Meier curves after noting the date of first visit, the date of last visit or death (for mortality), or the date of last follow-up or pulse loss (for those with pre-pulseless TAK for the assessment of eventual pulse loss). The date of initiation of disease-modifying anti-rheumatic drugs (DMARDs) and the date of change of first DMARD were used to calculate the duration of survival on the first DMARD. Data for the study was censored in October 2023.

Comparisons were performed between pre-pulseless TAK and TAK with pulse loss. Continuous variables were presented using means with standard deviations (SD) and compared using unpaired Student’s t test. Categorical variables were presented as percentages and compared using Chi-square test or Fisher’s exact test (if any of the four cells had values less than five). Clinical features, vascular involvement, and Hata’s angiographic subtypes at presentation were compared between pre-pulseless TAK and TAK with pulse loss using univariable logistic regression to generate odds ratios [OR, with 95% confidence intervals (CI)]. Multivariable-adjusted logistic regression models were constructed using those variables with a p value ≤ 0.1 on univariable logistic regression separately for clinical features, angiographic involvement, and Hata’s angiographic subtypes to compute adjusted odds ratios (aOR). These multivariable-adjusted regression models were adequately powered as there were at least fifty events of pre-pulseless TAK for analysis ^16^. Survival curves were compared using the log-rank test. Hazard ratios (HR, with 95%CI) for time-to-event outcomes between pre-pulseless TAK and TAK with pulse loss were calculated using the Cox proportional hazards assumption. The proportionality of hazards was statistically tested using p values for Shoenfeld residuals calculated using the “estat phtest” command on STATA. All the statistical analyses were conducted using STATA 16.1 I/C (STATA Corp, United States of America).

## RESULTS

From 245 patients with TAK in the cohort, 238 with angiographically-proven disease [of whom 91 (38.23%) had pre-pulseless TAK] were included in the analysis. The mean (±SD) duration of follow-up for the entire cohort was 43.8 (±48.7) months. Computed tomographic angiography and positron emission tomography-computed tomography were the most frequently used diagnostic angiographic modalities (**Supplementary Table S1**). TAK with or without pulse loss had similar age at disease onset, at diagnosis, at cohort entry, and similar delay to diagnosis. Overall, three-fourths of the patients with TAK in the cohort were female subjects, however, the proportion of male subjects was relatively higher in those with pre-pulseless TAK. Fewer patients with pre-pulseless TAK fulfilled the 1990 ACR classification criteria (≥3 points) or 2022 ACR-EULAR classification criteria (≥5 points). However, the 2022 ACR-EULAR classification criteria were fulfilled by a greater proportion of pre-pulseless TAK than the 1990 ACR classification criteria (18/91 vs 7/91, Chi square p value 0.018). TAK with pulse loss had higher proportions of patients with active disease at baseline by PGA, higher ITAS2010, and DEI.TAK scores, were more often initiated on corticosteroids or DMARDs than those with pre-pulseless TAK (**Table 1**). Methotrexate, mycophenolate, and tacrolimus were the most commonly used DMARDs in both groups (**Supplementary Table S2**). The use of biologic or targeted synthetic DMARDs in our cohort was sparse (**Table 1**). Anti-hypertensive medications were more frequently prescribed in pre-pulseless TAK. Similar proportions of patients in both groups underwent open surgical or endovascular interventions related to TAK. The proportion of deaths was similar in TAK with or without pulse loss (**Table 1**). Pre-pulseless TAK was associated with significantly lesser frequency of asymmetry of pulses or blood pressure [aOR 0.34, 95%CI 0.18–0.63), upper limb claudication (aOR 0.38, 95%CI 0.18–0.82), or lower limb claudication (aOR 0.28, 95%CI 0.12–0.68), and more frequently had deranged renal function (aOR 4.43, 95%CI 1.58–12.37). Pre-pulseless TAK were less likely to have right subclavian (aOR 0.45, 95%CI 0.23–0.90) or left carotid artery involvement (aOR 0.42, 95%CI 0.21–0.84) (**Table 2**). Hata’s angiographic subtype IV was more common in pre-pulseless TAK (aOR 8.02, 95%CI 2.61–24.65) (**Supplementary Table S3**).

**Table 1.** Characteristics of the cohort.

**Parameter**	**Pre-pulseless TAK (n=91)**	**TAK with pulse loss(n=147)**	p value*
Age at disease onset (Mean ± SD)	25.03 ± 10.94	25.33 ± 9.30	0.825
Paediatric-onset TAK [n(%)]	33 (36.26%)	38 (25.85%)	0.088^a^
Age at diagnosis (Mean ± SD)	28.00 ± 11.24	28.69 ± 10.46	0.629
Age at cohort entry (Mean ± SD)	28.95 ± 11.52	29.96 ± 10.95	0.497
Sex distribution (F:M)	57:34	115:32	**0.009^a^**
Diagnostic delay (years) (Mean ± SD)	3.06 ± 3.87	3.49 ± 5.10	0.492
Duration of follow-up (months) (Mean ± SD)	34.86 ± 43.86	47.62 ± 51.56	0.051
Fulfilled 1990 ACR classification criteria [n(%)]	73 (80.22%)	144 (97.96%)	**<0.001** ^b^
Number of items fulfilled on the 1990 ACR classification criteria (Mean ± SD)	3.55 ± 1.23	4.56 ± 1.02	**<0.001**
Fulfilled 2022 ACR/EULAR classification criteria [n(%)]	84 (92.31%)	144 (97.96%)	**0.047** ^b^
Number of items fulfilled on the 2022 ACR/EULAR classification criteria (Mean ± SD)	9.13 ± 3.52	11.73 ± 3.28	**<0.001**
Mortality (n, %)	2 (2.20%)	10 (6.80%)	0.138^b^
Baseline DEI.TAK (Mean ± SD)	5.49 ± 3.81	11.41 ± 6.22	**<0.001**
Baseline ITAS2010 (Mean ± SD)	5.98 ± 4.34	13.39 ± 7.01	**<0.001**
Active disease at baseline [n(%)]	57 (62.64%)	119 (80.95%)	**0.002^a^**
Initiated on corticosteroids	55 (60.44%)	118 (80.27%)	**<0.001** ^**a**^
Initiated on DMARDs^%^	52 (57.14%)	114 (77.55%)	**<0.001** ^**a**^
Total number of DMARDs	0.81 ± 0.89	1.17 ± 0.94	**0.004**
Received anti-hypertensives [n(%)]	79 (87.78%)	106 (72.11%)	**0.005^a^**
Received aspirin [n(%)]	18 (19.78%)	39 (26.53%)	0.236^a^
Received clopidogrel [n(%)]	8 (8.79%)	15 (10.20%)	0.720^a^
Received statins [n(%)]	10 (10.99%)	18 (12.24%)	0.770^a^
Underwent interventions	17 (18.68%)	24 (16.33%)	0.640^a^

* Unpaired t test for mean(SD),

Chi squared^a^/Fisher’s exact^b^ for proportions

% 74 individual DMARDs had been initiated in patients with pre-pulseless TAK (none on biologic or targeted synthetic DMARDs).

172 individual DMARDs had been initiated in patients with TAK with pulse loss (5 on biologic or targeted synthetic DMARDs). ACR: American College of Rheumatology; DMARDs; Disease-modifying anti-rheumatic drugs; DEI.TAK: Disease Extent Index in TAK; EULAR: European Alliance of Associations for Rheumatology; ITAS2010: Indian TAK Clinical Activity Score; SD: Standard deviation; TAK: Takayasu arteritis.

p values <0.05 are highlighted in bold

**Table 2.** Comparison between pre-pulseless TAK and TAK with pulse loss.

**Parameter**	**Univariable odds ratio (95% CI) (n = 238)**	**Multivariable-adjusted odds ratio (95% CI) (n = 238)**
**Clinical features at presentation**		

Constitutional features	1.16 (0.69 – 1.96)	-
Carotidynia	**0.28 (0.08 – 0.99)**	0.35 (0.09 – 1.32)
Headache	1.32 (0.71 – 2.45)	-
Syncope, dizziness, or vertigo	**0.45 (0.22 – 0.92)**	0.54 (0.25 – 1.20)
TIA or Stroke	0.78 (0.37 – 1.65)	-
Seizure	2.98 (0.85 – 10.48)	2.82 (0.75 – 10.73)
Blurring vision	1.27 (0.59 – 2.77)	-
Loss of vision	0.59 (0.15 – 2.29)	-
Pulse or BP inequality	**0.39 (0.23 – 0.68)**	**0.34 (0.18 – 0.63)**
Vascular bruits	0.79 (0.45 – 1.39)	-
Upper limb claudication	**0.23 (0.12 – 0.48)**	**0.38 (0.18 – 0.82)**
Lower limb claudication	**0.35 (0.17 – 0.75)**	**0.28 (0.12 – 0.68)**
Hypertension	**2.27 (1.09 – 4.74)**	2.12 (0.92 – 4.88)
Aortic regurgitation	0.89 (0.29 – 2.75)	-
Deranged renal function	**4.93 (1.97 – 12.35)**	**4.43 (1.58 – 12.37)**
Abdominal angina	2.52 (0.69 – 9.20)	-
Chest pain	0.83 (0.37 – 1.88)	-
Heart failure	0.87 (0.40 – 1.92)	-

**Arterial involvement**		

Brachiocephalic	**0.31 (0.16 – 0.62)**	0.67 (0.27 – 1.64)
Right subclavian	**0.30 (0.16 – 0.53)**	**0.45 (0.23 – 0.90)**
Left subclavian	**0.38 (0.22 – 0.66)**	0.69 (0.36 - 1.32)
Right carotid	**0.37 (0.21 – 0.66)**	1.07 (0.50 – 2.30)
Left carotid	**0.27 (0.15 – 0.47)**	**0.42 (0.21 – 0.84)**
Right vertebral	0.22 (0.03 – 1.84)	-
Left vertebral	**0.24 (0.08 – 0.70)**	0.38 (0.12 – 1.25)
Pulmonary artery	0.80 (0.26 – 2.41)	-
Coronary artery	3.28 (0.29 – 36.71)	-
Ascending aorta	0.59 (0.30 – 1.14)	-
Arch of aorta	0.59 (0.34 – 1.02)	1.27 (0.63 – 2.55)
Descending thoracic aorta	0.72 (0.42 – 1.21)	-
Abdominal aorta	1.39 (0.81 – 2.38)	-
Celiac trunk	1.46 (0.82 – 2.60)	-
Superior mesenteric artery	1.57 (0.88 – 2.81)	-
Inferior mesenteric artery	0.70 (0.21 – 2.36)	-
Right renal	**2.59 (1.51 – 4.43)**	1.91 (0.99 – 3.69)
Left renal	**2.10 (1.23 – 3.58)**	1.12 (0.58 – 2.20)
Right iliac	0.38 (0.11 – 1.40)	-
Left iliac	0.65 (0.22 – 1.92)	-
Right femoral^$^	-	-
Left femoral	0.81 (0.07 – 9.01)	-

$ Right femoral artery involvement excluded from the logistic regression model due to collinearity

p values <0.05 are highlighted in bold

95%CI: 95 percent confidence intervals; TAK: Takayasu arteritis; TIA: Transient ischemic attack.

**Figure 1.**
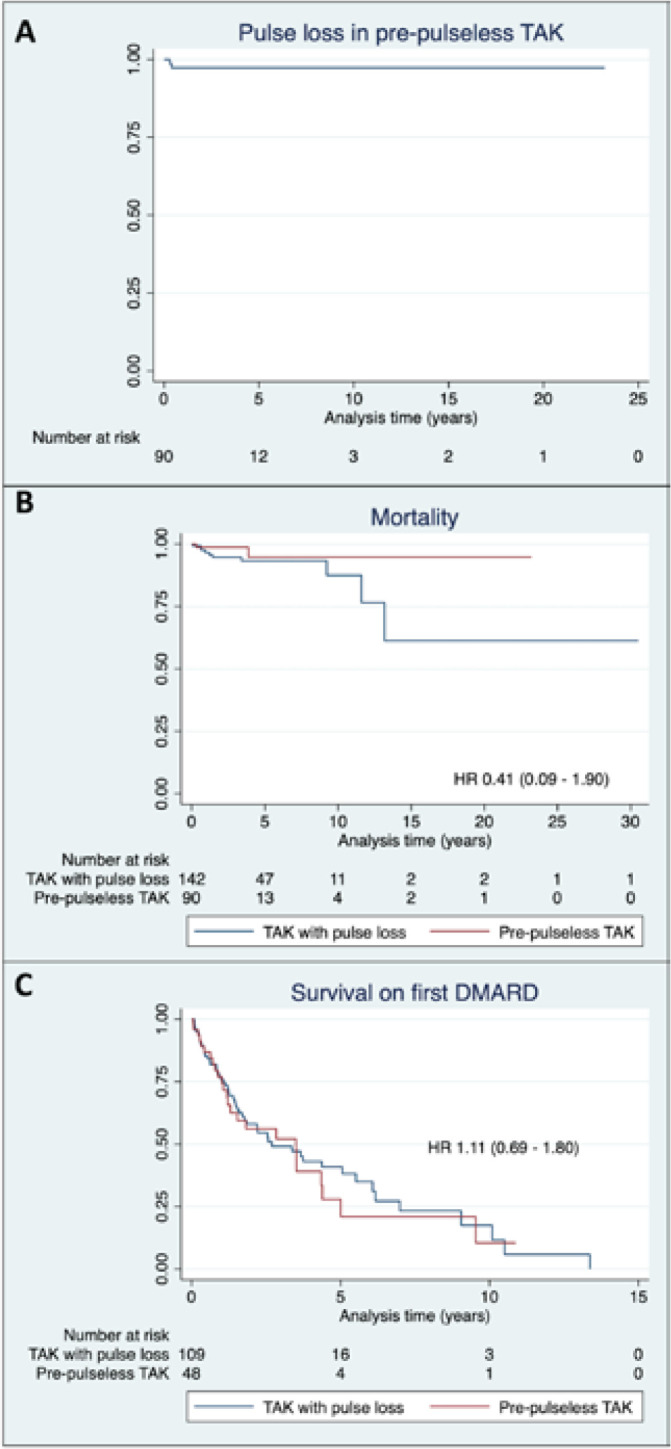
A: Kaplan-Meier curve for subsequent pulse loss in those with pre-pulseless Takayasu arteritis (TAK) at presentation. B: Comparison of mortality between TAK with or without pulse loss. C: Comparison of duration of survival on the first disease-modifying antirheumatic drug (DMARD) between TAK with or without pulse loss. HR: Hazard ratio (95% confidence intervals presented within brackets).

Only two patients with pre-pulseless TAK subsequently developed pulse loss on follow-up (both within six months of follow-up) (**Figure 1A**). The risk of mortality (HR 0.41, 95%CI 0.09 – 1.90, p value for Shoenfeld residual 0.763, log-rank test p value=0.242, **Figure 1B**) and survival on the first DMARD (HR 1.11, 95%CI 0.69–1.80, p value for Shoenfeld residual 0.972, log-rank test p value=0.658, **Figure 1C**) were similar for pre-pulseless TAK and TAK with pulse loss. Proportions of TAK with active disease on follow-up were similar between both the groups at 6 months, 1 year, 2 years, 5 years, and 10 years of follow-up (**Supplementary Table S4**).

## DISCUSSION

Compared with TAK with pulse loss, patients with pre-pulseless TAK were more likely to be male subjects, more frequently had deranged renal function and Hata’s type IV disease, and less often had asymmetry of pulse or blood pressure, limb claudication, subclavian or carotid artery involvement. Fewer patients with pre-pulseless TAK fulfilled the 1990 ACR classification criteria for TAK than TAK with pulse loss. Although pre-pulseless TAK had a lesser frequency of active disease and lower ITAS2010 and DEI.TAK scores at the initial assessment than TAK with pulse loss, similar proportions of patients in both groups had active disease on follow-up. Most patients with pre-pulseless TAK did not develop pulse loss on follow-up. Survival was similar in both groups.

The delay to diagnosis was similar in both groups. Only two patients with pre-pulseless TAK subsequently developed pulse loss. Both these patients developed pulse loss within six months of follow-up. Therefore, pre-pulse-lessness was likely not related to the temporal evolution of TAK. These findings reiterate the observations from the Vasculitis Clinical Research Consortium and National Institutes of Health cohorts of TAK from the United States of America that a triphasic pattern of disease is unusual in TAK.^8^

Criticism has been levelled against the 2022 ACR-EULAR TAK classification criteria for their reduced specificity in real-life settings.^17^ However, a greater proportion of pre-pulseless TAK fulfilled the 2022 ACR-EULAR classification criteria than the 1990 ACR classification criteria. This suggests that the 2022 ACR-EULAR classification criteria possibly capture a broader spectrum of TAK than the 1990 ACR criteria. Given the significantly lower 2022 ACR-EULAR criteria scores in pre-pulseless TAK than in TAK with pulse loss, it may be hypothesized that higher 2022 ACR-EULAR criteria scores indicate greater disease extent. TAK with pulse loss also had higher baseline ITAS2010 and DEI.TAK scores than pre-pulseless TAK, however, the prognosis was similar between both groups. The ITAS2010 and DEI.TAK have been criticized as more accurately reflecting disease extent rather than disease activity, which might also explain higher scores in TAK with pulse loss.^7^

A lower frequency of asymmetry of pulse or blood pressures, limb claudication, and subclavian or carotid artery involvement likely reflect the features anatomically linked to the absence of pulse loss in pre-pulseless TAK. Overall, abdominal aorta involvement and hypertension are more prevalent in TAK from Asia than elsewhere.^10,18^ A greater frequency of abdominal aorta involvement and renovascular hypertension resulting in deranged renal functions in pre-pulseless TAK possibly reflect the presence of manifestations other than pulse loss which required for a diagnosis of TAK to be made in these patients.

There were limitations to the present study. The information was derived from a monocentric cohort which limits the generalisability of the findings. However, TAK is a rare LVV, and the cohort included a large number of patients with TAK. While some of the data was retrieved retrospectively, this information was collected from patients following up in a dedicated vasculitis clinic with standardised data collection. Few patients were excluded for missing data. Longitudinal follow-up of the cohort for an extended duration enabled the confirmation of the fact that most patients with pre-pulseless TAK remain pre-pulseless over time. The large number of patients with pre-pulseless TAK permitted multivariable-adjusted logistic regression analyses for the associations of clinical and angiographic features with pre-pulseless TAK. However, the rarity of some of these manifestations such as deranged renal functions resulted in wide confidence intervals (thereby, greater uncertainty) of some of the odds ratios. To the best of our knowledge, this is the first systematic assessment of the presentation and prognosis of pre-pulseless TAK compared with TAK with pulse loss.

To conclude, patients with pre-pulseless TAK more often had abdominal aorta involvement and impaired renal functions. Pre-pulseless TAK was associated with a lesser frequency of limb claudication, asymmetry of pulse or blood pressure, and carotid or subclavian involvement. Despite these differences in phenotype, survival and long-term outcomes were similar in TAK with or without pulse loss.

## Figures and Tables

**Supplementary Table S1.** Angiographic modalities used for diagnosis of Takayasu arteritis in the cohort.

**Modality used for angiography**	Number (Percentage) (n=238) *
Positron emission tomography – computed tomography	107 (44.96%)
Computed tomographic angiography	183 (76.89%)
Magnetic resonance angiography	51 (21.43%)
Colour Doppler Ultrasound	33 (13.87%)
Conventional angiography	26 (10.92%)

* More than one angiographic modality could have been used in an individual patient.

**Supplementary Table S2.** DMARDs used in pre-pulseless TAK and TAK with pulse loss.

**Pre-pulseless TAK**	**TAK with pulse loss**
**First-line DMARD**	**First-line DMARD**
Methotrexate (n=18)	Methotrexate (n=46)
Mycophenolate (n=15)	Tacrolimus (n=44)
Tacrolimus (n=12)	Mycophenolate (n=15)
Azathioprine (n=5)	Azathioprine (n=5)
Cyclophosphamide followed by azathioprine (n=1)	Cyclophosphamide followed by azathioprine (n=1)
Tacrolimus + Methotrexate (n=1)	Cyclophosphamide (n=1)
	Methotrexate + Mycophenolate (n=1)
	Tocilizumab (n=1)
**Second-line DMARD**	**Second-line DMARD**
Methotrexate (n=5)	Mycophenolate (n=14)
Mycophenolate (n=5)	Azathioprine (n=10)
Tacrolimus (n=4)	Tacrolimus (n=6)
Azathioprine (n=1)	Methotrexate (n=4)
	Leflunomide (n=2)
	Adalimumab (n=1)
	Tocilizumab (n=1)

DMARD: Disease-modifying anti-rheumatic drug; TAK: Takayasu arteritis

**Supplementary Table S3.** Comparison of angiographic subtypes between pre-pulseless TAK and TAK with pulse loss.

**Parameter**	**Univariable Odds ratio (95% CI) (n = 238)**	**Multivariable-adjusted Odds ratio (95% CI) (n = 238)**
Hata’s I	0.69 (0.29 – 1.66)	-
Hata’s IIa	0.68 (0.17 – 2.71)	-
Hata’s IIb	**0.29 (0.10 – 0.88)**	0.36 (0.12 – 1.09)
Hata’s III	1.23 (0.41 – 3.66)	-
Hata’s IV	**8.82 (2.88 – 27.00)**	**8.02 (2.61 – 24.65)**
Hata’s V	0.84 (0.50 – 1.44)	-
Hata’s P+	0.80 (0.26 – 2.41)	-
Hata’s C+	3.28 (0.29 – 36.71)	-

p values <0.05 are highlighted in bold

95%CI: 95% confidence intervals; TAK: Takayasu arteritis

**Supplementary Table S4.** Proportion with active disease at different time-points compared between pre-pulseless TAK and TAK with pulse loss.

**Time point**	**Pre-pulseless TAK**	**TAK with pulse loss**	p value*
At presentation	57/91 (62.6%)	119/147 (81.0%)	**<0.001^a^**
6 months	7/58 (12.1%)	15/96 (15.6%)	0.541^a^
1 year	9/47 (19.1%)	16/78 (20.5%)	0.854^a^
2 years	4/26 (15.4%)	10/61 (16.4%)	>0.999^b^
5 years	2/11 (18.2%)	5/33 (15.2%)	>0.999^b^
10 years	0/3 (0%)	2/7 (28.6%)	>0.999^b^

* Chi squared^a^/Fisher’s exact^b^ for proportions

p values <0.05 are highlighted in bold

## Data Availability

All the analyses performed for this article have been reported in the main text or the supplementary files. Anonymised data pertaining to the article shall be shared on reasonable request to the corresponding author (Durga Prasanna Misra, durgapmisra@gmail.com)

## ABBREVIATIONS

95%CI: 95% confidence intervals
ACR: American College of Rheumatology
aOR: Adjusted Odds Ratio
DEI.TAK: Disease Extent Index in Takayasu Arteritis
DMARDs: Disease-modifying anti-rheumatic drugs
EULAR: European Alliance of Associations for Rheumatology
HR: Hazard ratio
ITAS2010: Indian Takayasu Arteritis Clinical Activity Score
LVV: Large vessel vasculitis
OR: Odds ratio
PGA: Physician global assessment
SD: Standard deviation
SGPGIMS: Sanjay Gandhi Postgraduate Institute of Medical Sciences
TAK: Takayasu arteritis
